# Effectiveness of a Web-Guided Self-Managed Telerehabilitation Program Enhanced with Outdoor Physical Activity on Physical Function, Physical Activity Levels and Pain in Patients with Knee Osteoarthritis: A Randomized Controlled Trial

**DOI:** 10.3390/jcm13040934

**Published:** 2024-02-06

**Authors:** Maria Moutzouri, George A. Koumantakis, Michael Hurley, Aggeliki Georgia Kladouchou, George Gioftsos

**Affiliations:** 1 Department of Physiotherapy, University of West Attica, 12243 Athens, Greece; gkoumantakis@uniwa.gr (G.A.K.); gioftsos@uniwa.gr (G.G.); 2 Centre for Allied Health, St George’s University of London, Cranmer Terrace, London SW17 0RE, UK; m.hurley@kingston.ac.uk; 3 Department of Rehabilitation Sciences, Kingston University, Holmwood House, Grove Crescent, Kingston upon Thames KT1 2EE, UK; 4 Orthopaedic Research UK, Furlong House, 10a Chandos Street, London W1G 9DQ, UK

**Keywords:** home-based, web-based, self-managed exercise, advice, physical activity, knee osteoarthritis

## Abstract

**Background**: Telerehabilitation to guide self-management has been shown to be a feasible care strategy for knee osteoarthritis (KOA). The aim of this study was to explore the effectiveness of a blended web-based rehabilitation program enhanced with outdoor physical activity (BWR-OPA) and consultation versus an OPA (usual care) program in KOA patients. **Methods**: Forty-four KOA participants were prescribed to follow the programs five times/week for 6 weeks. The primary outcome was self-reported physical function, measured by the Knee Injury and Osteoarthritis Outcome Score (KOOS). The secondary outcomes were pain, PA, function (timed up-and-go (TUG) test, 30 s chair rise test (30 s CRT)), psychological functioning and QoL. **Results**: There was a significant difference between the groups’ KOOSs for pain and symptom subscales at the 6- and 12-week post-intervention assessments compared to baseline (*p* < 0.005) favoring the BWR-OPA group. There was a superior improvement in PA in the BWR-OPA training group (*p* < 0.05). Statistical and clinical improvements were found (*p* < 0.001) with effect sizes over 2.0 for objective measures of function. Similar improvements were recorded over time (*p* < 0.005) at 12 weeks for QoL, KOOS subscales for ADL, QoL and sports/recreation and psychological functioning for both groups. **Conclusions**: A blended web-based self-managed care program with outdoor PA was superior in many respects to usual care in KOA participants.

## 1. Introduction

Knee osteoarthritis (KOA) is an incurable progressive disease with a global prevalence of ~23% in people over 40 years of age [1]. The social, professional and psychological impact of KOA is substantial, thus causing a marked decline in quality of life (QoL) [2]. The National Institute for Health and Care Excellence (NICE) [3] recommends that first-line treatment for KOA should include disease orientation and a long-term exercise program. There is high-quality evidence demonstrating the effectiveness of education and exercise in improving function and pain in individuals with KOA. However, long-term, routine face-to-face consultations for chronic diseases such as KOA are not feasible or cost-effective, and prescribed exercise remains underutilized or used in the short term [4,5,6]. The Osteoarthritis Research Society International (OARSI) [7] emphasizes the importance of self-management strategies among the central therapeutic approaches. Self-management can improve patients’ awareness of the disease and their overall health perception as well as boost patients’ confidence in managing symptoms [8,9]. Relevant studies have shown improvement in pain, stiffness and QoL but not physical function [10,11]. Limited access to healthcare services due to the aging population, obesity and restrictions of KOA volume promote the need to innovate in how treatment is provided [4,12,13].

Web-based technologies to guide self-management may be potential innovative and feasible alternative rehabilitation strategies given that people with KOA are anxiously seeking information about their condition from internet sources [14,15,16]. Web-based OA management programs consisting of exercises, informational sessions (including advice on, e.g., pacing, pain relief and the importance of physical activity (PA)) and outcome monitoring have shown beneficial findings [17,18]. However, the available sources often lack quality, evidence, clarity and long-term motivational strategies [19,20]. Some high-quality free online OA self-management platforms are available through healthcare organizations; however, they may only provide general information with no consultation or oversight [20]. Adherence to exercise is found to be poor in people with KOA, potentially explaining the lack of long-term clinical benefits of exercise [21,22]. Thus, self-care management strategies motivated by asynchronous remote treatment may be an efficacious and flexible alternative to be promoted. In addition to exercise and informational material, low- to moderate-intensity PA is considered essential, as a reflection of sustained behavioral change adoption in a more active and less fearful engagement of self-management. Perhaps the inclusion of real-life PA in the recommended exercise regimen may enhance comprehension and feasibility in adherence [23]. The knowledge gap that this study aims to address is whether the combination of an acknowledged web-based management program enhanced with real-life PA is beneficial for altering pain and behavior towards ADL when offered in a self-managed mode. 

Given the above, there is a need for a blended program that encompasses physical and behavioral elements, with purposeful and flexible PA as well as interplay of self-management and healthcare support strategies to promote a good fit for the care of KOA. This study primarily aims to compare the efficacy of a 6-week web-based rehabilitation program enhanced with outdoor structured PA and advice to self-manage pain and physical function in KOA patients compared to an outdoor PA program alone; secondarily, maintenance of the outcomes at mid-term (3-month follow-up period) is examined.

## 2. Methods

The current study was an assessor-blinded, parallel group, 2-arm prospective randomized controlled trial prospectively registered in the ISRCTN clinical trial registry (ISRCTN12950684/27-09-2020, https://www.isrctn.com/ISRCTN12950684, accessed on 29 November 2023). The protocol aligned with the Consolidated Standards of Reporting Trials (CONSORT) guidelines [24,25]. Ethics approval for the trial was granted by the Ethics Committee of the University of West Attica, Greece (49238/09-07-2020).

The study protocol has been thoroughly published elsewhere [26]. Digital informed consent was obtained using an online form prior to baseline assessments.

### 2.1. Study Participants

Study participants were recruited from the West Attica region of Athens in Greece from September 2020 to October 2021 via the following: (i) online advertisements placed in their municipalities, community centers, local newspapers, West Attica University and Facebook; (ii) brochures and study posters placed in medical and physiotherapy clinics of the region; and (iii) presentations about KOA conducted in the community and Peristeri KEP Ygeias (the local Centre for Health Exercise and Medicine). Screening was via community physiotherapists (not involved in the main research), and eligibility was confirmed by telephone. Broad inclusion criteria were used.

The inclusion criteria were as follows:

1. clinical (clinical criteria: age ≥ 45 years, activity-related knee pain and morning knee stiffness ≤ 30 min) or radiographic diagnosis of knee OA;
2. knee pain on most days for 3 months or more;
3. average overall knee pain severity of 4 or greater on an 11-point numeric rating scale (NPRS) during the previous week;
4. own a smart phone device or tablet;
5. home internet access;
6. ability to consent, participate and complete assessments.

The exclusion criteria were presented in the published study protocol [26].

### 2.2. Study Design and Procedures

#### 2.2.1. Randomization, Allocation Concealment and Blinding

Participants were randomized in a 1:1 ratio. Computer-generated randomization was performed by an external statistician. After baseline assessment with an MSc PT student (AK), participants were provided an envelope according to the randomization sequence by a volunteer undergraduate student who prepared consecutively numbered, sealed and opaque envelopes. The envelopes were kept in a locked location accessible only by the unblinded main researcher-physiotherapist (MM). This physiotherapist (MM) then scheduled the participants’ first face-to-face appointment to teach the rehabilitation program. Participants read and signed the informed consent sheet as per the Helsinki Declaration guidelines, providing details of the study’s aim to investigate a range of digital resources (e.g., computer and cell phone) to promote knee pain self-management.

#### 2.2.2. Study Groups

Both study arms relied on OA self-management programs that included web-based material and outdoor PA at pre-specified walks in journeys offered via maps (distance 600–900 m, at their own pace). The maps were created by MM based on the PA guidelines for this clinical population in accordance to the NICE guidelines for duration of PA [3,27]. The journeys were created in different safe spots (wide pavements and benches for rest) within the local city containing shopping malls, parks, historical monuments, open air theatres and in general places that may attract participants’ interest. Adherence was encouraged by weekly phone calls by the physiotherapist during the study period in both groups. Management of all participants’ knee and coexistent medical problems continued as per the primary care physician’s discretion but was recorded in a diary and documented at all assessment sessions.

Intervention Group: The treatment to the intervention group consisted of a 6-week prescribed program of combinatory elements executed in a self-managed manner. The blended web-based rehabilitation enhanced with outdoor PA (BWR-OPA group) composed of three elements: (1) a web-based structured video exercise program (twice weekly) and (2) KOA disease consultatory video sessions (once weekly), all based on the awarded ESCAPE-pain program [28,29] delivered digitally via a website. Thirdly, they were encouraged to follow an outdoor PA walk journey, as the control group, with a difference in the prescribed frequency, which was 3 times/week (instead of 5 times/week for the control group). Therefore, the prescribed volume of exercise was relatively equivalent, all following an exercise or PA program 5 times/week. The video-instructed exercises focused on neuromuscular leg strengthening, functionality and balance enhancement, as exemplified by doing sit-to-stand and stair-climbing exercises. The consultatory sessions covered the basics of OA, its treatment, self-managing symptoms, the benefits of behavioral change, pacing, goal setting, PA and maintaining a healthy lifestyle. The website was accessed via http://westwalks.uniwa.gr (accessed on 29 November 2023). Access was free of charge, and the general advice material was completely available; however, the video-based exercise and consultatory sessions were accessed using specific passwords that were given to the participants of the intervention group in the introductory face-to-face session. All participants received the same standardized website and were permitted to access it at will for 12 weeks.

Control group: The patients in the control group (OPA) received usual care, including general web-based information and advice for KOA and were encouraged to follow outdoor PA 5 times/week. They had access to the same website as the BWR-OPA group but only to the general recommendations (i.e., KOA pathology, importance of exercise and meeting PA guidelines) freely accessible to all.

#### 2.2.3. Outcomes

Outcomes were both patient-reported (PROMs) and performance-based objective measures (PBOMs). The outcomes were reliable and valid and well recommended for KOA clinical trials [30]. The primary outcomes were (a) physical function in the past week assessed by the Knee Injury Osteoarthritis Outcome Score (KOOS) Likert version, which is a disease-specific instrument with good psychometric properties demonstrated in a range of OA studies [31] that includes 42 items in 5 separately scored subscales, namely, pain, other symptoms, ADL, function in sport and recreation (sport/rec) and knee-related QoL, and (b) average knee pain over the previous week as measured with the Numerical Pain Rating Scale (NPRS). The NPRS consists of an 11-point scale with terminal descriptors of no pain (score 0) and extreme pain (score 10) and is highly reliable, valid and responsive to change in this clinical population [32]. All participants were monitored similarly and had a face-to-face meeting at enrollment (i.e., baseline), after 6 weeks and after 12 weeks (i.e., end of study) to assess pain, function, PA levels and QoL. 

The secondary outcomes included the between-group differences in change in the two patient objective outcomes (PBOMs) of physical functioning (i.e., the 30 s sit-to-stand test and the timed up-and-go [TUG] test). Both are validated methods presenting acceptable reliability (ICC > 0.7) for assessing participants’ ADL-relevant physical function [33,34]. The 30 s sit-to-stand test was assessed as the number of times the participant could rise from a sitting position in a chair to a full standing position in 30 s, and the TUG test was assessed as the time (measured in seconds) required for the participant to stand up on a therapist’s command, walk 3 m, turn around, walk back to the chair and sit down again [33]. At enrollment, participants had a demonstration of the tests and practiced once before doing each test. The TUG test was repeated 3 times, and the mean time was used. The 30 s sit-to-stand test was conducted once only to avoid fatigue effects. A break was allowed between both tests. 

Assessment of quality of life was performed using the Short-Form 12 (SF-12), which has been found as psychometrically sound for this clinical population [35]. Habitual PA levels were recorded according to (1) a self-reported diary, (2) the Modified Baecke Physical Activity Questionnaire (mBQ), validated in samples of adults ≥55 years of age to assess PA [36] and positive ratings of reliability for knee osteoarthritis patients [37] and (3) the Lower Extremity Activity Scale (LEAS) for lower limb osteoarthritis [37]. This scale is a lower limb disease-specific PA scale for which patients select the most representative of their daily living category of the 12 statements given and has been found as valid and reliable for KOA patients [37]. Psychological function was measured using the Tampa Scale of Kinesiophobia (TSK), with satisfactory validity and reliability in KOA patients [38], to investigate the fear of pain related to painful or harmful activities, acknowledged as an important cognitive factor in relation to chronic pain and disability, anxiety, depression and effectiveness of treatment in knee OA patients [39]. 

Adverse events were participant-reported at 12 weeks, defined as any problem believed to be caused by the study intervention requiring treatment or medication and/or interfering with function for 2 days or more. 

Exercise adherence was assessed by the number of days/week and time spent (in min) performing walks and knee exercises during the previous week recorded in a diary. Diaries were collected at 6 and 12 weeks.

#### 2.2.4. Data Analysis

The effects of the blended rehabilitation program were assessed for each outcome measure using separate factorial analyses of variance (ANOVAs) involving group (intervention; control) by test occasion (baseline, 6 weeks post-intervention and 12 weeks post-intervention) comparisons, with repeated measures on the latter factor. Assumptions underpinning the use of ANOVA were assessed and corrections used (Greenhouse–Geisser (GG)) when appropriate. Analyses were performed by using the statistical package for social sciences (IBM SPSS, version 24.0). Repeated measures analysis of variance (ANOVA) was performed for all primary and secondary outcome measures. The effect size (ES; Cohen’s *d*) was calculated for independent groups using pooled standard deviations [40].

A clinically meaningful difference in the primary outcome of physical function measured by the KOOS was considered to be 15%. The margin was derived from a minimal important difference KOOS-pain subscale (MCID_80_ KOOS-PS) score of 10 units reported for the KOOS based on the study by Lyman [41] in KOA patients. A sample size of 22 participants per group was computed with (GPower software, version 3.1.9.7) to achieve an experimental design sensitivity of 0.80 for the KOOS (Type I and Type II error rates, 0.05 and 0.20, respectively) in discriminating a moderate relative ES between the performance of the groups at the study’s primary endpoint (12 weeks post-intervention) [41]. Assuming a 15% loss to follow-up, 50 participants were recruited in the study. Statistical significance was accepted at *p* < 0.05. 

## 3. Results

**Figure 1:**
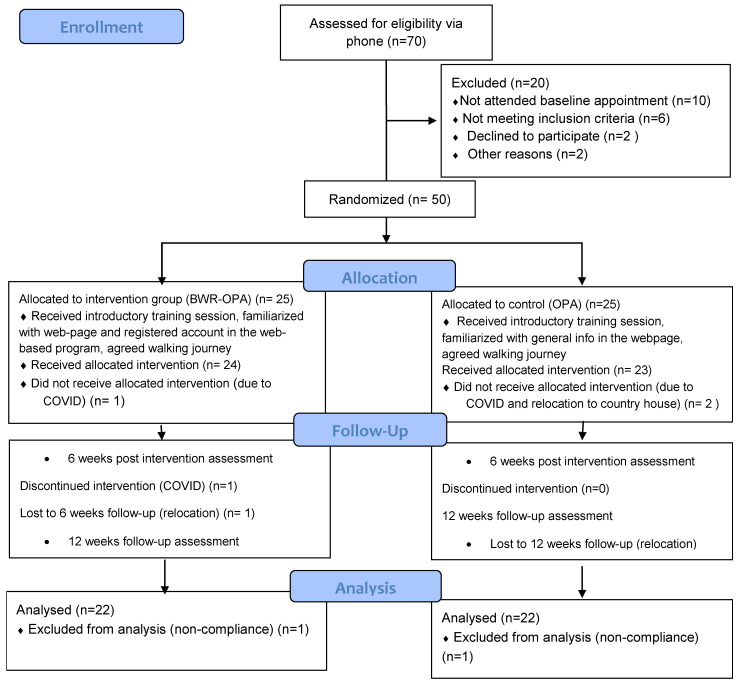
Study’s CONSORT flow chart.

The control and intervention participant CONSORT flowchart, including exclusions and losses to follow-up, is shown in Figure 1. Among a total of 70 participants screened for eligibility, 50 were randomized, including 25 participants randomized to the intervention group and 25 participants randomized to the usual care group. Ten participants did not attend the baseline appointment, and no baseline data or demographic characteristics were obtained for these participants. Owing to the COVID-19 lockdown in Greece and associated relocations to participants’ country houses to leave the city center, three participants in the usual care group and three participants in the intervention group were lost to follow-up (Figure 1) and were therefore excluded from the analyses. A total of 44 participants were analyzed, namely, 22 participants in the intervention group (mean [SD] age, 65.1 [5.3] years; 15 [68.2%] women; 13 [59.0%] retired; and mean [SD] BMI, 24.1 [5.5]) and 22 participants in the control group (mean [SD] age, 63.5 [5.6] years; 19 [86.3%] women; mean [SD] BMI, 23.9 [5.9]; and 15 [68.2%] retired) who completed this study. 

No significant difference was observed between participant groups in terms of demographic characteristics, pain, physical or PA level at baseline (Table 1). Relating to the compliance with the prescribed time of exercise and/or PA, the mean (SD) adherence with the web-based exercise program was ~70% compared with ~48%.

The intervention group yielded superior gains in self-reported physical function and pain compared to the control group. These gains in physical function for the intervention group ranged between 30% for the TUG test (*p* < 0.05) and 32.5% for the 30s CRT (vs. 18.3% for the control group) (*p* < 0.05) by the end of follow-up. Table 2 shows group mean scores for the intervention and control groups at baseline, 6 weeks and 12 weeks post-treatment. Comparisons using a priori orthogonal difference contrasts suggested that the superior gains made by the intervention group for the TUG test were elicited progressively over the period of training, with gains elicited between baseline and 6 weeks post-treatment (%) and between 6- and 12-weeks post-treatment (%). These were similar in magnitude but significantly greater than the control values (*F* > 4.8; *p* < 0.05; Table 3).

### 3.1. Primary Outcome

No between-group analysis of the mean change from baseline to 6 weeks was shown for the KOOS subscales; however, the intervention group improved statistically significantly over the 12-week time for the KOOS subscales of pain (*F* = 11.9; *p* < 0.001) and symptoms (*F* = 8.9; *p* < 0.005). Moreover, statistically significant differences were shown for both groups for the KOOS subscales of ADL (*F* = 13.8; *p* < 0.01), sports (*F* = 14.6; *p* < 0.001) and QoL (*F* = 11.8; *p* < 0.001) over the 12 weeks. The intervention group showed a statistically significant greater decrease in the NPRS pain score from baseline to 6 weeks, which was maintained over 12 weeks compared to the control group (*F* = 4.3; *p* < 0.05).

### 3.2. Secondary Outcomes

With regards to the PBOMs, statistically significant differences were found between groups for the TUG test (between-group difference, −2.0 [95% CI, −1.3 to −3.3]; *F* = 4.8; *p* < 0.05) and the 30 s chair stand test (between-group difference, 3.8 [95% CI, 2.6 to 7.9] *F* = 4.0, *p* < 0.05), with the intervention group showing greater improvement. 

**Figure 2:**
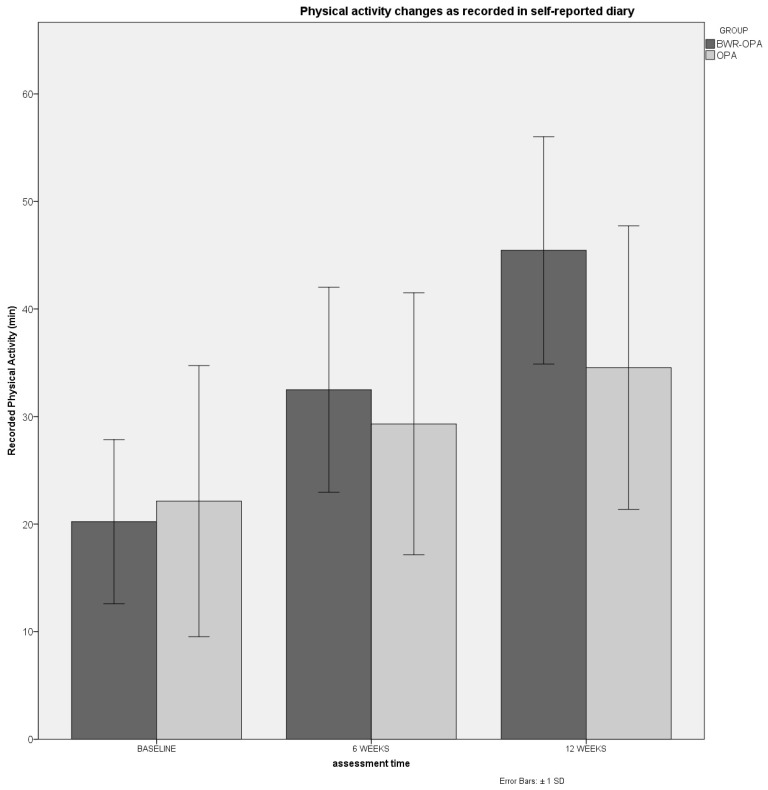
Progression of self-reported PA as recorded in a weekly diary.

Statistically significant between-group differences were found for PA measures, with the LEAS results showing greater improvement in the intervention group at 6 weeks, which was maintained over the 12 weeks of follow-up (*F* = 7.3; *p* < 0.01). According to the mBQ scale, only the sport subscale of PA showed statistically significant greater improvement in the intervention group compared to the control group at 6 weeks (*F* = 3.8; *p* < 0.05). Within-group differences were found to be statistically significant for the total mBQ scale for the intervention group (*F* = 6.3; *p* < 0.01), indicating superior PA levels over time. Both groups showed a higher level of PA over 12 weeks for the subscale of work (*F* = 10.6; *p* < 0.01) and leisure (*F* = 21.6; *p* < 0.001). Concerning the diary of weekly recorded PA, statistically significant differences were found between groups over time, with the intervention group showing superior levels of PA (*F* = 4.3; *p* < 0.05) (Figure 2).

There were no statistically significant between-group differences regarding changes in QoL measures (*F* = 0.17; *p* > 0.05). Both the SF-12 physical and mental components showed statistically significant within-group improvements for the two groups at 12 weeks of follow-up (*F* = 9.9; *p* < 0.005). Moreover, within-group changes in TSK scores only for the intervention group were found at 12 weeks (*F* = 20.2; *p* < 0.001). 

No serious adverse events were reported in any of the study groups.

## 4. Discussion

The current study of a blended approach for self-managed web-based exercise, consultation and prescribed real-life outdoor PA compared to usual encouragement of PA showed a statistically significant reduction and clinically meaningful improvement in pain and in physical performance clinical tests. The self-reported pain and symptom subscales, recorded for the KOOS, in the intervention group showed statistically significant and clinically important improvements at the 12-week endpoint time, as the improvements over the current study go beyond the MCID of the total KOOS (8–10 units) in KOA patients. As far as the remaining subscales of the KOOS, namely, ADL, sports/rec and QoL, both groups showed statistically significant improvements over time. Health care professionals often advice patients to follow general PA as the least means of self-managing KOA; thus, the control group in the current study was encouraged to undertake regular PA and have access to generic information on KOA. 

With regards to functional mobility as measured with the recommended OARSI clinical tests [33], TUG test and 30s CRT, statistically significant and clinical improvements (as MCID for the TUG test: 0.8–1.4 and MCID for the 30s CRT: 2–3 receptions in OA research) were shown for the intervention versus the control group. The blended exercise group exhibited a double-sized (~30%) improvement in the TUG test and 30s CRT compared to the control group (12.5–18.5%) improvement. The only web-based telerehabilitation study using PBOMs for measuring functional mobility did not show statistically significant improvements in the aforementioned clinical tests [42]. However, in the study by Allen et al. [42], the comparator groups referred to face-to-face physiotherapy visits and web-based PT visits and therefore it was important to observe that a web-based exercise program was not inferior to the usual direct access physiotherapy sessions.

Self-reported PA as recorded with the LEAS and the individual weekly diary showed statistically significant greater increases in the blended group compared to the control group. According to the mBQ PA scale, the intervention group elicited greater improvements in the sport subscale. Moreover, the total mBQ score showed statistically significant gains in PA level over time only for the blended exercise group. However, both groups showed significant gains over time in the PA level for the subscales of work and leisure. According to a recent systematic review [43], two published studies [42,44] have investigated the PA levels with web-based exercise programs; only the study by Bossen et al. [44], which actually focused on PA with the patients’ preferred activity, has elicited statistically meaningful increases in PA levels. Levels of PA in older adults have been significantly associated with levels of functionality, levels of KOA severity and QoL [45]. Therefore, it is considered essential to encourage specific PA within rehabilitation programs via both exercise and comprehension pathways, as the current study has encapsulated.

Regarding the secondary outcome measures, statistically significant improvements in pain (as measured with the NPRS and KOOS subscale for pain) and kinesiophobia (as measured with the TSK) were recorded between groups, with the blended exercise group showing superior improvements. With regards to pain, the minimally important clinical difference in the NPRS is set at 30% (2 points) for chronic pain conditions, which in the intervention group of the current study was achieved at the end of the program at the 6-week (2.1 points difference) and 12-week follow-up times (~3.0 points difference). Only within-group differences were found for QoL (as measured with SF-12) and the KOOS subscales for ADL, sports/rec and QoL for both groups, showing that behavioral changes towards PA or exercise can ameliorate health-related mobility and QoL. Importantly, both groups’ improvements exceeded the MCID (4.5 point) set for this clinical population.

A recent systematic review [43] included five relevant web-based studies of medium-to-high methodological robustness (PEDro > 6–8/10), involving exercise (with a main focus on muscle strengthening and aerobic activity) showing statistically significant improvements in pain (3/5 studies) and function (2/5 studies). Three of the five web-based studies incorporated exercise combined with education (with elements of cognitive behavioral therapy and self-management strategies on KOA), and all found statistically significant improvements in pain and function in KOA patients [46,47,48]. With regards to PA according to the systematic review by Moutzouri et al. [43], three studies [49,50,51] delivering tele-rehabilitation with telephone/text messaging and one web-based telerehabilitation study [52] showed statistically significant improvements in the PA levels of KOA patients.

To our knowledge, this is the first study of a blended web-based strategy with minimal oversight over weekly phone calls to encompass (1) exercise, (2) self-management strategies and (3) enhanced outdoor PA for KOA patients. The program was delivered in a self-determined time and offered all central tenets of clinical guidelines for KOA patient care, i.e., evidence-based exercise, education for pain coping and cognitive behavioral skills to boost participants’ confidence in dealing with their condition as well as enhanced outdoor PA. The contribution of the established award-winning program ESCAPE knee pain, which does not require specialized training, sophisticated exercises or equipment for the participants, in the rehabilitation regime of the intervention group has assured quality control of the study. Moreover, the combination of online support with the provision of video exercise and consultation, with even a minimal face-to face or telephone guidance as well as the inclusion of real-life outdoor activity has perhaps enhanced patients’ adherence [23,53,54]. A study by Nelligan et al. [55] was of a similar self-directed nature and contained similar tenets, but no oversight was offered, just automated texts to enhance compliance and no face-to-face first training for participants. The study also showed improved pain and self-reported function but not PA levels [55]. The outdoor PA journeys in the current study were created exclusively for each municipality in order to attract participants’ interest by combining walks with recreation. The wide spectrum of selected outcome measures, some recommended for clinical trials of OA [33], covering clinically important concepts is important for the clinical interpretation of the study’ findings. Finally, the novelty of the study is that the blended program was conducted in weak structured areas where cost-effective options of physiotherapy care are mostly needed.

On the other hand, within the limitations of the study is the unblinded nature of the physiotherapist delivering intervention and guidance. In some cases, accessibility limitations emerged due to the restrictions imposed by COVID-19, and the above were addressed with telecommunication. Another limitation was that PA levels were not measured objectively with activity monitors, with the risk of bias due to the fact that patients based their response on cognitive/memory skills [56]. This study piloted the delivery of ESCAPE-pain resources in Greece in a relevantly weak municipality of West Athens, and therefore it was delivered to a small number of participants limiting generalizability of the findings. Although the volume of exercise and/or PA amongst groups was prescribed with caution to be relatively equivalent (both groups having physical exercise 5 times/week for 30–40 min), the nature of the ESCAPE-pain resources supplies participants with support holistically. Therefore, the intervention group in the current study was offered this guidance in comparison with the control group that was given a realistic usual care with general advice and activity, thus preventing comparability of programs. As this was a community-based study, broad clinical criteria were used for study inclusion regarding diagnosis of KOA (clinical and/or radiological), so there was a lack of comparability between groups regarding the degree of KOA. 

## 5. Conclusions

This study showed that a 6-week blended approach of prescribed self-managed web-based exercise, consultation and enhanced PA provided superior clinical benefits for pain, physical performance and PA levels compared to usual encouragement of outdoor PA. Self-managed care delivered in this blended manner with minimal oversight could be an effective option for KOA patients to achieve improved phycological function and relevant good adherence levels. It is strongly advised for people with KOA to engage in low to moderate-intensity PA, as both groups showed improvements in QoL over time in the 3-month follow-up. Future studies are suggested to include KOA participants from both urban and agricultural areas in a multicenter trial to assure generalizability as well as investigate the level of adherence more thoroughly both online and objectively via reliable pedometers.

## Figures and Tables

**Table 1:** Participant demographic and clinical characteristics.

		BWR-OPA Mean (SD), n = 22	OPA Mean (SD), n = 22
Age (years)		65.1 (5.3)	63.5 (5.6)
Weight (kg)		74.7 (19.1)	77.0 (12.7)
Sex (%)	Female	86.4	68.2
	Male	13.6	31.8
Occupation (%)	Housewife	3.0 (13.6)	1.0 (5.9)
	retired	13.0 (59.1)	15.0 (68.2)
	worker	6.0 (27.3)	6.0 (27.3)
Pain NPRS (cm)		5.5 (0.9)	5.8 (0.8)
TUG (S)		11.1 (1.4)	11.2 (1.9)
PA (LEAS)		8.6 (1.6)	9.1 (1.7)
PA (DIARY) (MIN)		22.3 (10.1)	22.1 (16.2)

BWR-OPA: Blended web-based rehabilitation-outdoor physical activity; SD: standard deviation; NPRS: Numeric pain rating scale; TUG: Timed up-and-go test; PA: Physical activity; and LEAS: Lower Extremity Activity Scale.

**Table 2:** Comparison of PROMs of pain, functionality and PA in both intervention and control groups.

	Variable	Group	Baseline	6th Week	*p* Value	ES Cohen’s *d*	12th Week	*p* Value	ES Cohen’s *d*
Pain	NPRS	BWR-OPA	5.5 (0.8)	3.4 (0.8)	ns	2.6	2.4 (1.3)	0.04	3.8
		OPA	5.8 (0.9)	4.0 (1.2)		2.0	3.2 (1.1)		2.8
	KOOS-P	BWR-OPA	28.6 (17.9)	60.0 (15.5)	ns	1.6	76.1 (14.5)	0.001	2.7
		OPA	32.3 (21.8)	61.0 (14.8)		1.3	66.9 (12.3)		1.6
Functionality	KOOS-S	BWR-OPA	58.8 (16.5)	66.7 (16.6)	ns	0.5	70.9 (14.8)	0.005	0.7
		OPA	60.2 (16.8)	66.2 (16.8)		0.4	68.4 (18.5)		0.5
	KOOS-ADL	BWR-OPA	58.6 (14.4)	66.0 (18.3)	ns	0.5	70.9 (17.1)	ns	0.9
		OPA	61.3 (20.4)	66.0 (22.0)		0.2	68.5 (20.6)		0.4
	KOOS-SP	BWR-OPA	28.6 (17.9)	36.5 (17.4)	ns	0.4	41.3 (15.6)	ns	0.7
		OPA	32.3 (21.8)	40.0 (26.8)		0.4	40.8 (26.8)		0.4
	KOOS-QoL	BWR-OPA	42.1 (18.7)	48.6 (18.0)	ns	0.3	58.8 (14.6)	ns	0.9
		OPA	42.8 (19.1)	48.1 (22.1)		0.3	53.7 (17.3)		0.6
PA	LEAS	BWR-OPA	8.6 (1.6)	11.7 (2.8)	0.001	1.9	13.5 (1.5)	0.001	3.0
		OPA	9.1 (1.7)	10.1 (1.7)		0.6	10.2 (1.8)		0.6

BWR-OPA: blended web-based rehabilitation-outdoor physical activity; ES: effect size; NPRS: Numeric Pain Rating Scale; KOOS-P: Knee Osteoarthritis Outcome Score pain subscale; KOOS-S: symptom subscale; KOOS-ADL: everyday living subscale; KOOS-SP: sports and recreation subscale; and LEAS: Lower Extremity Activity Scale.

**Table 3:** Progression of PBOMs of functionality for the timed up-and-go test and the 30s CRT for the intervention and control groups over time.

PBOMs	Group	Baseline	6th Week	*p* Value	ES Cohen’s *d*	12th Week	*p* Value	ES Cohen’s *d*
TUG test (s)	BWR-OPA	11.1 (1.4)	9.3 (1.3)	0.001	1.3	7.8 (1.0)	0.001	2.4
	OPA	11.2 (1.9)	10.4 (2.0)		0.4	9.8 (1.9)		0.7
30s CRT (n)	BWR-OPA	16.4 (3.9)	21.4 (5.5)	0.001	1.3	24.2 (5.1)	0.001	2.0
	OPA	16.5 (4.8)	18.1 (4.0)		0.3	19.1 (4.7)		0.5

PBOMs: objective outcome measures, TUG: timed up-and-go test; 30s CRT: 30 s chair rise test; BWR-OPA: blended web-based rehabilitation-outdoor physical activity; and ES: effect size.

## Data Availability

No new data are created. Supporting data availability upon request.
